# Malarial proteases and host cell egress: an ‘emerging’ cascade

**DOI:** 10.1111/j.1462-5822.2008.01176.x

**Published:** 2008-06-17

**Authors:** Michael J Blackman

**Affiliations:** Division of Parasitology, National Institute for Medical ResearchMill Hill, London NW7 1AA, UK

## Abstract

Malaria is a scourge of large swathes of the globe, stressing the need for a continuing effort to better understand the biology of its aetiological agent. Like all pathogens of the phylum Apicomplexa, the malaria parasite spends part of its life inside a host cell or cyst. It eventually needs to escape (egress) from this protective environment to progress through its life cycle. Egress of *Plasmodium* blood-stage merozoites, liver-stage merozoites and mosquito midgut sporozoites relies on protease activity, so the enzymes involved have potential as antimalarial drug targets. This review examines the role of parasite proteases in egress, in the light of current knowledge of the mechanics of the process. Proteases implicated in egress include the cytoskeleton-degrading malarial proteases falcipain-2 and plasmepsin II, plus a family of putative papain-like proteases called SERA. Recent revelations have shown that activation of the SERA proteases may be triggered by regulated secretion of a subtilisin-like serine protease called SUB1. These findings are discussed in the context of the potential for development of new chemotherapeutics targeting this stage in the parasite's life cycle.

## Introduction

All apicomplexan pathogens spend part of their life cycles within the confines of a host cell or cyst. *Plasmodium*, the causative agent of malaria, is no exception. At different points in its life cycle the parasite finds itself residing within either an oocyst on the basal side of the female *Anopheline* mosquito mid gut wall, a vertebrate hepatocyte (or fibroblast) or a red blood cell (RBC). In the latter two cases the parasite is not free within the host cell cytosol, but lies within a membrane-bound parasitophorous vacuole (PV). Following a period of repeated mitotic replication – the length and extent of which varies considerably depending on species and life cycle stage – the parasite exits from the host cell in a process known as egress. Many details of how the parasite makes its escape are obscure, but the accumulated evidence indicates an important role for parasite proteolytic enzymes. This review aims to critically summarize this evidence in the expectation that it may provide plausible future strategies in the search for improved control of malaria and other pathologies caused by apicomplexan parasites. As a full appraisal of the molecular role of proteases in egress cannot logically be considered in isolation from the ultrastructural and physicomechanical aspects of the process, this article comprises three sections: first, it briefly reviews what is known about the mechanics of malaria parasite egress; second, it examines the biochemical and genetic evidence implicating proteases in egress; and finally it attempts to draw these together into a collective model of how egress may be regulated and targeted. Throughout, the main focus is on egress of *Plasmodium falciparum* merozoites from the blood-stage schizont, because of its particular significance for chemotherapeutic control of severe clinical malaria and because the majority of experimental work on egress has been on this developmental stage.

## The mechanics of egress

**Fig. 1:**
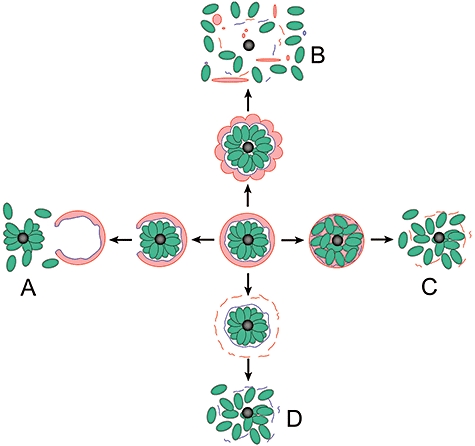
Current models of asexual blood-stage merozoite egress.A. The membrane fusion model of Winograd *et al*. (1999).B. The ‘explosive rupture’ model of Glushakova *et al*. (2005) in which the schizont forms a short-lived ‘flower’ structure.C. The ‘inside-out’ model (Wickham *et al*., 2003) in which PVM breakdown precedes RBC membrane rupture.D. The ‘outside-in’ model in which the RBC membrane is degraded first, allowing release of merozoites surrounded by the PVM, which is eventually degraded in an exoerythrocytic step (Salmon *et al*., 2001; Soni *et al*., 2005).

Live video microscopy has played a special role in studies on egress. Real-time light microscopic visualization of *in vitro* blood-stage egress and events leading up to it in live parasites has been performed in the avian parasite *Plasmodium lophurae* (Trager, 1956), in the simian parasite *Plasmodium knowlesi* (Dvorak *et al*., 1975) and in *P. falciparum* (Winograd *et al*., 1999; Wickham *et al*., 2003; Glushakova *et al*., 2005). In all cases it was observed that egress is a rapid – and therefore, by inference, highly regulated – event. Some debate exists over the details, and the various studies have led to a number of different if not necessarily mutually exclusive models for merozoite egress. These will not be discussed in detail here, but they condense to the following four schemas. In the first (Fig. 1A), merozoites are released in a cluster through a single opening in the schizont, leaving behind a membranous ‘ghost’ comprising the breached PV membrane (PVM) and RBC membrane (Winograd *et al*., 1999). Because the residual cytosol of this structure was observed to persist for an extended period after merozoite release, it was proposed that the initial opening is likely a result of localized fusion of the PVM and RBC membrane, effectively creating a large pore to allow parasite exit. The second model (Fig. 1B), which can almost be viewed as the diametrically opposite scenario, is that egress is quite literally an explosive event, in which the mature parasitized cell undergoes a short-lived increase in intracellular volume before its sudden rupture, scattering the free merozoites away from the site of rupture and leaving behind vesiculated fragments of the separate host cell and PV membranes (Glushakova *et al*., 2005). The third model, referred to here as the ‘inside-out’ model, proposes that egress involves two distinct stages in which degradation of the PVM occurs prior to RBC membrane rupture (Fig. 1C). Evidence for this derives from the use of transgenic *P. falciparum* clones stably expressing green fluorescent protein (GFP) either exclusively in the PV lumen or in the RBC cytosol. Irrespective of its previous location, the GFP marker was observed to relocalize to fill the entire infected cell late in schizogony (Wickham *et al*., 2003), interpreted as a result of PVM breakdown just prior to eventual egress. In the last model, referred to here as the ‘outside-in’ model, a two-stage process is again postulated. Here, however, the first step in egress is disruption of the RBC membrane (Fig. 1D), releasing clusters of merozoites still surrounded by an intact PVM which is subsequently degraded in an exoerythrocytic event (Salmon *et al*., 2001; Soni *et al*., 2005). Importantly, these PVM-enclosed merozoite structures (PEMS) were detected by Salmon *et al*. at low frequency in regular *P. falciparum* cultures, suggesting that they represent a normal, short-lived egress intermediate. This last model is not directly supported by real-time microscopic evidence, but by other data described below.

Electron microscopy (EM)-based ultrastructural analyses cannot detect subtle time-dependent effects, but they have a place in investigating the mechanistic basis of egress. Both Langreth *et al*. (1978) and Aikawa (1971) noted that intact, highly mature blood-stage schizonts occasionally lack a discrete PVM, consistent with the above-described ‘inside-out’ model of PVM degradation preceding RBC membrane rupture at egress. Immuno-EM analysis of mature wild-type parasites with antibodies to the PV-resident protein S-antigen reached a similar conclusion (Wickham *et al*., 2003); once again, it was noticed that this protein relocalized to fill the entire luminal space of the infected cell in mature schizonts.

It will be clear from the above that it is presently difficult to draw hard and fast conclusions regarding the temporal sequence of PVM and RBC membrane rupture at egress. Intuitively, it would seem unlikely that blood-stage egress should differ fundamentally from egress of other intracellular stages; after all, why should the malaria parasite invent the wheel twice? In this context, although relatively little is known about the mechanics of egress in other life cycle stages, recent elegant work by Heussler and colleagues has revealed some important parallels between egress of blood-stage and liver-stage merozoites. Hepatocytes infected *in vivo* or HepG2 cells infected *in vitro* with GFP-expressing transgenic clones of the rodent malaria *Plasmodium berghei* eventually round up, detach from neighbouring cells and die. This is concomitant with disappearance of the PVM and release of the mature merozoites into the host cell cytosol (Sturm *et al*., 2006; Sturm and Heussler, 2007). This observation, very clearly detectable in these relatively large cells, is again consistent with the ‘inside-out’ model for blood-stage egress described above.

## Egress requires protease activity

Whatever the physicomechanical details of egress and the temporal sequence of PVM and host cell membrane rupture, all except perhaps the fusion model invoke a requirement for generalized destabilization of the two membranes and host cell cytoskeleton to allow their eventual disruption. This notion has encouraged a search for parasite-derived molecular mediators of egress. The earliest documented evidence suggesting protease involvement in egress came from a series of studies indicating effects on egress and RBC invasion by *P. knowlesi* upon treatment with a range of protease inhibitors including the broad-spectrum serine and cysteine protease inhibitors leupeptin and chymostatin (Banyal *et al*., 1981; Dutta and Banyal, 1981; Hadley *et al*., 1983). Lyon and Haynes (1986) showed that the presence of a mixture of leupeptin, chymostatin, antipain (a serine and cysteine protease inhibitor) and pepstatin (an aspartic protease inhibitor) in cultures of *P. falciparum* asexual blood stages resulted in the accumulation of mature schizonts in which the normal process of egress was blocked. Analysis by SDS-PAGE and Western blot of these stalled forms, referred to as ‘protease inhibitor clusters of merozoites’ (PCM), revealed defects in the proteolytic processing and shedding of a range of parasite proteins, including fragments of a major schizont surface protein now known as merozoite surface protein-1 (MSP1). Shortly after this study, Delplace *et al*. (1988) demonstrated that leupeptin alone could prevent *P. falciparum* schizont rupture, concomitantly interfering with the proteolytic processing of an abundant PV protein called P126 (now known as SERA5 – see below). Examination by EM of the stalled schizonts showed that, whereas rupture of the RBC membrane was prevented by the leupeptin treatment, disintegration of the PVM had taken place, releasing the mature merozoites into the residual cytoplasm of the host cell. This important finding provided the first evidence that rupture of the PVM and RBC membranes is differentially sensitive to protease inhibitors. Several more recent studies have built on that work. Salmon *et al*. (2001) showed that formation of PEMS in cultures could be substantially upregulated by the presence of the epoxide-based cysteine protease inhibitor E64, interpreted as indicating that PVM but not RBC membrane rupture involves an E64-sensitive protease. Removal of the E64-mediated block (by washing) showed that the PEMs contained viable, invasive merozoites. As E64 is effectively an irreversible inhibitor, this is an unexpected finding, but was interpreted by the authors as indicating continued biosynthesis of the cysteine protease(s) targeted by the E64, eventually overcoming the block in egress. A study by Soni *et al*. (2005) reached a virtually identical conclusion, again demonstrating that the presence of either leupeptin or E64 produced extracellular clusters of merozoites apparently surrounded by a still-intact PVM. Using their GFP-expressing clones, Wickham *et al*. (2003) effectively replicated and extended the finding of Delplace and colleagues; they showed that a mixture of leupeptin and antipain allowed PVM rupture while blocking RBC membrane rupture, whereas E64 treatment selectively blocked PVM rupture but allowed RBC membrane degradation. In a different approach, Gelhaus *et al*. (2005) found that treatment of schizonts with biotinylated dibenzyl aziridine-2,3-dicarboxylate, an irreversible cysteine protease inhibitor, prevented egress by blocking rupture of the RBC membrane, though apparently allowing PVM rupture. Sturm and Heussler (2007), in their examination of liver-stage merozoite egress from *P. berghei*-infected HepG2 cells, found that treatment of the cells with E64 blocked egress and clearly prevented the PVM degradation that usually precedes egress in this system.

It will be evident that the above studies reach sometimes conflicting conclusions, although much of the contrary data (e.g. the different effects of leupeptin in different studies) could in part be a result of experimental variables such as different culture conditions and differences in the age of the RBC used in the cultures. Notwithstanding this, at least three common threads can be drawn from the accumulated data. These are: that breakdown of the PVM and host cell membrane are differentially regulated; that both events are protease-dependent; and that PVM rupture is an E64-sensitive process. Given the high specificity of E64 for cysteine proteases, this strongly implicates one or more cysteine proteases in PVM rupture, and at least one additional distinct activity in host cell membrane rupture. What are the likely identities of the responsible proteases, and how are their activities regulated?

## Candidate proteases mediating egress

### A dual function for haemoglobinases?

Three general approaches have been taken to establish the identity of proteases participating in egress: analysis of the subcellular localization and substrate diversity of known malarial proteases; gene disruption studies; and the use of selective small compound inhibitors. Much of the interest in malarial proteases has been driven by the phenomenon of haemoglobin digestion by the blood-stage parasite. While it is still unclear whether the primary role of this huge catabolic effort is to provide a source of amino acids for the growing parasite or simply to make space for it inside the host cell, interfering with it is detrimental to parasite growth. Thus far, four *P. falciparum* aspartic proteases – plasmepsins I, II, III (otherwise known as histoaspartic protease, or HAP) and IV, plus three papain-like cysteine proteases called falcipain-2, -2′ and -3 – have been implicated in haemoglobin digestion. Digestion takes place within an acidic digestive vacuole (see Rosenthal, 2002, for a review). The finding that, in addition to its haemoglobinase activity, plasmepsin II can also digest the host RBC cytoskeletal proteins spectrin, protein 4.1, and actin at neutral pH, and that the protease can be detected in mature schizonts within the cytoplasm of the infected RBC (Le Bonniec *et al*., 1999), raised the possibility that plasmepsin II might be involved in host cell membrane destabilization at egress. Similarly, falcipain-2 was demonstrated to be capable of digesting ankyrin and protein 4.1 at neutral pH (Dua *et al*., 2001; Hanspal *et al*., 2002), and a peptide based on the cleavage site in ankyrin inhibited the activity of recombinant falcipain-2 and also inhibited parasite maturation and egress when delivered into schizonts (Dhawan *et al*., 2003). In the same study, falcipain-2 expression was detected in subcellular locations external to the parasite, both in the PV and in vesicular structures extending into the RBC cytosol. These findings are all provocative, but need to be assessed in the light of subsequent gene disruption studies that have shown that neither plasmepsin II nor falcipain-2 is essential in asexual blood stages, and the knockout lines exhibit no egress phenotype (Omara-Opyene *et al*., 2004; Sijwali and Rosenthal, 2004; Liu *et al*., 2005; 2006; Sijwali *et al*., 2006). Furthermore, there is no evidence that specific inhibitors of aspartic proteases (such as pepstatin) inhibit egress when used alone, and other gene disruption experiments have demonstrated that plasmepsins I, IV and HAP, as well as falcipain-1 and 2′, are all individually dispensable in asexual blood stages. It is too early to rule out a role for any of these enzymes in egress but the indications are that, if they are involved, there must be a degree of redundancy in the system. Finally, it remains unclear how the activities of these enzymes might be controlled to achieve the very tight temporal regulation implied by the light microscopic studies described above, although an endogenous inhibitor of falcipains has been identified that could conceivably play a role in this (Pandey *et al*., 2006).

### The enigmatic SERA family: PV-located potential mediators of egress

The full power of reverse genetics was demonstrated by a beautiful study on the role of a different putative protease in egress of midgut sporozoites from oocysts in the mosquito vector. In an investigation of genes previously shown to be upregulated during *P. berghei* midgut sporozoite biogenesis, Aly and Matuschewski (2005) examined the consequences of disruption of a gene they named egress cysteine protease 1 (*ecp1*). As expected, the knockout lines exhibited no phenotype during blood-stage growth and the early stages of sporogony, but the resulting midgut sporozoites failed to egress from oocysts. Phase microscopic examination of the *ecp1(−)* oocysts showed that the trapped sporozoites moved in a continuous circular motion inside the intact oocysts, in contrast to mature wild-type oocyst sporozoites which adopted a radially oriented, mostly motionless state within the oocysts. Mechanical dissection of the *ecp1(−)* oocysts showed that the freed sporozoites possessed normal gliding motility but were completely non-infectious when mechanically transmitted to mice, in contrast to wild-type oocyst sporozoites which were infectious (albeit poorly compared with the salivary gland sporozoite stage that is naturally transmitted to the vertebrate host). The oocyst wall is a two-layered structure, with an inner wall of parasite origin and an outer, thicker wall derived from the basal lamina of the insect midgut epithelium. Further examination of the *ecp1(−)* oocysts showed that they were structurally different from wild-type oocysts, being refractory to permeabilization with saponin. Although the subcellular site of expression of ECP1 was not determined, and ECP1 has not been demonstrated to possess protease activity, the authors speculated that it might play a role in degradation of the inner wall of the oocyst at egress. Consistent with this, comparison by Western blot of *ecp1(−)* and wild-type oocysts detected a difference in the pattern of proteolytic processing of the circumsporozoite protein (CSP), a dominant component of the inner oocyst wall.

The above seminal findings – which proved that oocyst rupture is not simply a passive consequence of parasite replication – have profound implications for egress in other life cycle stages. The *P. falciparum* orthologue of *ecp1*, called *SERA8*, belongs to a family of nine genes, eight of which (*SERA1–8*) are disposed in a tandem head-to-tail array on chromosome 2, while *SERA9* is on chromosome 9. All the SERA gene products share a central relatively conserved papain-like domain as well as N- and C-terminal regions that contain a number of conserved Cys residues (Miller *et al*., 2002). The first identified family member, now known as SERA5, was originally named P126, P140 or P113, because of its apparent molecular mass on SDS-PAGE, or SERP or serine-rich antigen because of the presence of a stretch of up to 37 consecutive Ser residues near its N-terminus. SERA5 was first described as an abundant PV-localized protein that appeared in *P. falciparum* culture supernatants in a proteolytically truncated form upon schizont rupture (Delplace *et al*., 1985; Delplace *et al*., 1987), and that could induce antibodies that either protected against blood-stage infection *in vivo* (e.g. Perrin *et al*., 1984; Delplace *et al*., 1988; Inselburg *et al*., 1991; Enders *et al*., 1992) or interfered with egress or invasion *in vitro* (Chulay *et al*., 1987; Lyon *et al*., 1989; Sugiyama *et al*., 1996; Fox *et al*., 1997; Pang and Horii, 1998; Pang *et al*., 1999). Expression levels of the different family members varies considerably in blood stages, with *SERA5* and *SERA6* (originally called *SERP H*; Knapp *et al*., 1991) being the mostabundantly transcribed and translated (Aoki *et al*., 2002; Miller *et al*., 2002); indeed, proteomic analyses have indicated that SERA5 is among the most abundant schizont-stage parasite proteins (Lasonder *et al*., 2002; Le Roch *et al*., 2004). Although all the *SERA* genes are transcribed in asexual blood stages (Aoki *et al*., 2002; Miller *et al*., 2002), gene-targeting experiments have shown that most are dispensable; only *SERA5* and *SERA6* could not be disrupted, suggesting these are essential in this life cycle stage (Miller *et al*., 2002; McCoubrie *et al*., 2007). Of particular interest, while SERA6, 7 and 8 (cysteine-type) possess a Cys residue at the position of the canonical catalytic Cys, SERA1–5 and 9 (serine-type) have a Ser at this position (Kiefer *et al*., 1996) and appear to form a distinct phylogenetic group (Hodder *et al*., 2003; Bourgon *et al*., 2004), perhaps suggesting a distinct function. Consistent with this, although the total number of SERA genes differs in different *Plasmodium* genomes, all those that have been examined to date appear to contain at least one each of the two SERA types (Hodder *et al*., 2003; McCoubrie *et al*., 2007). An as yet unanswered question is the significance of this catalytic residue substitution; replacement with Ser of the active-site Cys in papain or cathepsin L abolishes enzyme activity (Clark and Lowe, 1978; Coulombe *et al*., 1996), so on this basis it might be predicted that the serine-type SERA proteins are proteolytically inactive. On the other hand, one subfamily of papain-like proteins, the silicateins, are demonstrably hydrolases and also possess the Cys-to-Ser substitution found in the serine-type SERA subgroup (Shimizu *et al*., 1998; Cha *et al*., 1999; Muller *et al*., 2007; 2008). Furthermore, a recombinant, refolded form of the SERA5 papain-like domain shows weak chymotrypsin-like activity (Hodder *et al*., 2003). It remains likely that both the serine- and cysteine-type SERA members do indeed mediate proteolytic roles in the parasite.

### SERA processing is temporally associated with egress

**Fig. 2:**
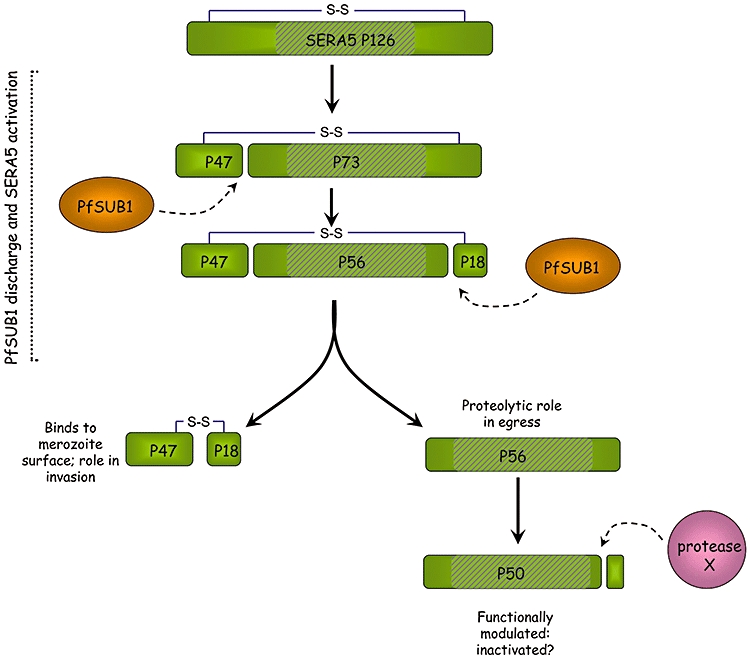
Model of SERA5 processing in the moments just prior to *P. falciparum* asexual blood-stage egress. The SERA5 P126 precursor is located in the PV. Regulated discharge of PfSUB1 results in cleavage at two sites, releasing P56 which contains a central papain-like domain (hatched) to play a proteolytic role in egress. The terminal P47 and P18 products of PfSUB1 processing remain in a disulfide-bonded complex (blue line indicates disulfide bridge) and bind to the merozoite surface. P56 is eventually truncated by an unknown cysteine protease to modulate its function, perhaps by inactivation. PfSUB1 likely processes SERA4 and SERA6, and perhaps other blood-stage SERA family members, in a similar manner.

Independent of the *P. berghei ecp1* study described above, a number of observations on *P. falciparum* SERA biology have triggered extensive speculation regarding a possible role in promoting egress and subsequent invasion. First, all the *SERA* gene products that have been detected in blood stages – SERA3, 4, 5, 6 and possibly 9 – are most highly expressed at schizont stage and localize to the PV lumen (Delplace *et al*., 1987; Knapp *et al*., 1989; 1991; Aoki *et al*., 2002; Miller *et al*., 2002), putting them in the right place and at the right time to partake in egress. Second, early studies noted a remarkably close temporal association between the proteolytic processing of SERA5 and blood-stage egress, with several observing that whereas processed products of the SERA5 precursor (referred to here as SERA5 P126) are abundant in culture supernatants following schizont rupture, only the precursor could be detected in unruptured schizonts. In contrast, only unprocessed SERA5 P126 was released upon mechanical rupture of mature schizonts (using saponin or freeze–thaw), indicating that SERA5 processing requires naturally mediated egress and is not simply a spurious consequence of non-specific release of degradative parasite proteases into culture medium accompanying egress (Delplace *et al*., 1985; 1987; 1988). This important point is returned to below. Detailed examination of SERA5 processing, including N-terminal sequence analysis of affinity-purified processed products, showed that processing takes the form of an initial cleavage at a site just N-terminal to the papain-like central domain, followed by a second cleavage C-terminal to it (Fig. 2) (Debrabant *et al*., 1992; Li *et al*., 2002a,b). The released central fragment, called P56, is then further processed by C-terminal truncation to the terminal P50 form which eventually accumulates in culture supernatants. In contrast, the N- and C-terminally derived processing fragments, called P47 and P18, respectively, remain associated in a disulfide-dependent manner and appear to bind to the surface of the released merozoites (Li *et al*., 2002b; Okitsu *et al*., 2007). This intriguing observation is supported by the finding that antibodies to P47, but not to P50, interfere with RBC invasion by the released merozoites (Sugiyama *et al*., 1996; Fox *et al*., 1997; Pang *et al*., 1999); this may explain the *in vivo* protection observed in the many SERA5 immunization trials referred to above. One provocative interpretation of these findings is that SERA5 – and perhaps the other SERA family members, as they all share a similar overall structure – is a dual-function protein, with a central domain that acts as a soluble protease and plays a role in egress, and terminal domains that interact with partner proteins on the merozoite surface and play a quite separate role in merozoite viability following egress (Fig. 2).

### SERA processing by SUB1: an activation event?

Most proteases are synthesized as inactive zymogens that are converted to their active forms by proteolytic cleavage, either in an autocatalytic intramolecular manner or *in trans*. It therefore seems plausible to predict that SERA5 processing represents an activation event. In an elegant attempt to characterize the protease(s) responsible, Li *et al*. (2002a) examined parasite extracts for proteolytic activity capable of converting a recombinant form of SERA5 P126 to polypeptide fragments resembling the authentic initial P47 and P73 processing products. Such an activity was detected; it was most abundant in schizont stages, it was membrane-associated, requiring a non-ionic detergent for efficient extraction, and it was sensitive to diisopropyl flurophosphate (DFP) but not to a range of other protease inhibitors including another broad-spectrum serine protease inhibitor, phenylmethylsulfonyl fluoride (PMSF). This implicated it as an unusual serine protease. Importantly, the recombinant SERA5 P126 used for these experiments was stable in the absence of parasite extracts, indicating it was incapable of autocatalytic processing. Further investigation showed that a different parasite activity might be responsible for conversion of P73 to the P56 and P18 products (though these data were less clear), while a third distinct, leupeptin- and E64-sensitive protease was clearly involved in truncation of P56 to P50 (consistent with the leupeptin sensitivity of this step noted by Delplace *et al*., 1988).

These findings have been greatly clarified by a recent study in which processing of SERA5 was shown to be mediated by a subtilisin-like serine protease called PfSUB1. Previous work had shown that this protease is expressed in a membrane-associated form in schizont stages, and had partly characterized its substrate preference and inhibitor profile using recombinant PfSUB1 (Blackman *et al*., 1998; Sajid *et al*., 2000; Withers-Martinez *et al*., 2002). Using a transgenic parasite line expressing epitope-tagged PfSUB1, Yeoh *et al*. (2007) showed that PfSUB1 was expressed in an unusual set of dense granule-like organelles (dubbed exonemes) from which it is released, in a fully soluble form, into the PV space just prior to egress. A selective PfSUB1 inhibitor prevented egress and also blocked SERA5 processing, suggesting a link between these events. Further examination showed that recombinant PfSUB1 correctly converted purified, parasite-derived SERA5 P126 into the P56 product, and an analysis of the processing sites using multiple alignments and synthetic peptide substrates demonstrated that PfSUB1 is also likely capable of processing most of the other SERA family members at equivalent sites; consistent with this, partially purified parasite SERA4 and SERA6 were also processed by recombinant PfSUB1 (Yeoh *et al*., 2007). In a separate recent study (Arastu-Kapur *et al*., 2008), a chloroisocoumarin selected in a screen for irreversible cysteine and serine protease inhibitors that selectively blocked egress was found to covalently label a major parasite protein that was identified as mature PfSUB1. A different egress-inhibitory compound, a dipeptide vinyl sulfone, covalently labelled a distinct parasite protease, identified as the cathepsin C-like dipeptidyl peptidase 3 (DPAP3). However, inhibition of this appeared to affect maturation or trafficking of PfSUB1 and other secreted parasite proteins, so this may have been an indirect effect on PfSUB1 expression. As PfSUB1 has the unusual property of being insensitive to a number of broad-spectrum serine protease inhibitors, including PMSF (Withers-Martinez *et al*., 2002), it can safely be concluded that the serine protease activity described above in the Li *et al*. (2002a) study in fact corresponds to PfSUB1. The ‘membrane-associated’ characteristics of PfSUB1 in its exoneme-resident form may be a result of these organelles being relatively detergent-resistant. Taken together with all the above data indicating a temporal link between SERA5 processing and egress, these findings raise the remarkable possibility that egress may be triggered by regulated discharge of PfSUB1 into the PV to activate multiple members of the SERA family. PfSUB1 appears to be an essential gene in blood stages, with orthologues in all *Plasmodium* species examined, and the above findings indicate it is a ‘druggable’ target. Interestingly, SERA orthologues were detected in a recent proteomic analysis of liver-stage schizonts (Tarun *et al*., 2008), so there is a distinct possibility that the above postulated egress pathway may also operate for this life cycle stage.

Why is SERA5 P56 truncated to P50 following the PfSUB1 processing step? The fact that this is blocked by leupeptin under conditions where RBC membrane rupture is prevented (Delplace *et al*., 1988) raises the possibility of a causal connection between these phenomena. Proteolytic inactivation of protease activity is well documented (e.g. Samis *et al*., 2000; Crawley *et al*., 2005), so if conversion of SERA5 P126 to P56 is an *activation* event then could conversion of P56 to P50 represent a subsequent *inactivation* step or other functional modification associated with RBC membrane rupture? This is just one more unanswered question regarding this intriguing proteolytic pathway. Identification of the cysteine protease responsible would be a first step in addressing it.

## Lessons from other intracellular pathogens and conclusions

Many questions about malarial egress remain unresolved, not least the fundamental issue of how proteases can promote destabilization of the PVM and RBC membranes. Simplistically, two broad mechanisms of protease action may be imagined; proteases may act as indirect regulators of egress by activating distinct effector proteins or signal transduction pathways. Alternatively, proteases may directly mediate the membrane and cytoskeletal destabilization presumably required for egress, by hydrolysis of integral membrane or cytoskeletal proteins as described above for plasmepsin II and falcipain-2. Other obligate intracellular pathogens may provide clues: several intracellular bacteria and *Leishmania* use pore-forming proteins or phospholipases for egress, and *Chlamydia* egress is a two-stage process in which the first step, breakdown of the intracellular inclusion vacuole, is cysteine protease-dependent (Hybiske and Stephens, 2007; 2008). Rather little is known of the role of proteases in egress of other apicomplexan genera, including the highly experimentally tractable *Toxoplasma gondii*. It is likely that future advances in these areas will shed useful light on malarial egress.

In conclusion, the present indications are that egress of different life cycle stages the malaria parasite involves mechanistically conserved pathways and the action of at least three essential proteases in the vertebrate host. Arguably, all these represent viable future targets for drugs designed to interfere with the critical step in the parasite life cycle. The task now is to enhance our understanding of this pathway and exploit it in new approaches to malaria control.
